# Late Action of the Georgia Medical Association Touching the Publication of Its Actions, and What Others Think of It

**Published:** 1885-06-20

**Authors:** 


### EDITORIALS AND MISCELLANEOUS.

### LATE ACTION OF THE GEORGIA MEDICAL ASSOCIATION TOUCHING THE PUBLICATION OF ITS TRANSACTIONS, AND WHAT OTHERS THINK OF IT.

In our last issue we made some remarks opposing the late action of the Georgia Medical Association in reference to publishing the transactions in a Medical Journal of this city, in which we stated that the action was unwise and unfortunate, and that we believed that it would be so regarded by the profession at large when properly understood. We are able to say that our prediction is already verified in the unsolicted correspondence of our office, and by certain of our exchanges outside of the State—in one of which the action of the Association is criticised severely.

So far as the interest of our Journal is concerned, we believe that in the end, we shall be benefitted rather than damaged ; for it is not to be supposed that fair and disinterested members of the profession at large will approve that which is so manifestly unjust and illiberal. Nor is it likely to comport with their principles or feelings to be told that, in order to get the transactions, they must take the Atlanta Journal, or any other Journal. Such a requirement will probably be regarded as a species of dictation which few will relish, and which will very naturally and properly be resented.

But the question as to whether or not this unfair discrimination against a leading journal will injure that journal does not, perhaps, so much concern the profession as the injury which is likely to result to the Association itself, and to the harmony of the profession in the State.

We are well aware that the occasional reference in our pages to the illiberality of the internal management of the Association did not set well with some of those who were working the wires in that body, but we can truly say that we have never denounced, nor sought to damage, the Association or profession in our State, but have endeavored to inculcate correct principles and impartiality in its management. Both in the Association and in the Legislature of Georgia the editors of this Journal have always sought to uphold the honor and dignity of the profession in the State. To this course we were bound, not only by an individual sense of duty, but by the fact that our Journal was established years ago as the friend and organ of the masses of the profession in our section.

What we have said heretofore was prompted by an honest opposition to wrong in the Association and in the profession, and we promise in the future to adhere firmly and boldly to the same policy.

We feel assured that the large majority of the members of the Association, not present when the late action was taken, will disapprove of it, and that the sober, second thought of those who did it will see and regret the mistake.

We forbear to say more on this matter, but will ask our readers to read the following sensible and forcible remarks by the intelligent editor of the Southern Clinic, published at Richmond, Va:

[From the Southern Clinic.]

### DISCRIMINATION WITH A VENGEANCE

We notice in the Atlanta Medical and Surgical Journal that at the last meeting of the Medical Association of Georgia the following report of the Committee on Publication was adopted:

"The Committee recommends that the Association accept the proposition of the Atlanta Medical and Surgical Journal, yiz.: To publish the transactions in the Journal for one year, furnish each member with the Journal and pay all expenses of the Secretary and Treasurer's office (except their expenses to and from the meetings) for $2.00 per member."

Here we see at a glance that, by the vote of this Association, there is an *official subscription* of every member of the Georgia Medical Association to the Atlanta Medical and Surgical Journal! And this is not all; it is arranged that a considerable amount of original matter from the more prominent members of the Georgia profession shall be furnished this journal to keep it well supplied ; so that, really, the Association throws its whole weight and influence, all of its funds, original papers and *individuality* into the Atlanta Medical and Surgical Journal.

We have no doubt that the Association flatters itself that it has acted wisely, and possibly the publisher of the Journal congratulates himself on this enterprising and business move, which gives him the winning hand over and against other reputable and equally deserving medical publications in the same State.

But from personal observation and upon general principles we are free to say, that nothing could prove more disastrous to the well-being and success of both the Association and journalism in Georgia, than this unwise, unjust and exceedingly illiberal action on the part of the Association.

The bitterest feeling that ever was engendered among the profession in Virginia was due to the fact that the Medical Society of Virginia annually published its transactions in a medical journal here, and lent its aid and influence to that journal, when there was another published here having an equal claim upon the profession for support and recognition. The final result was an overthrow of the clique and a publication of the transactions in separate and distinct form by contract. But not until many of the best members had become alienated and disgusted and left the Society forever.

Such action by any Association, where more than one publication exists, will inevitably bring about ill-will, discord, hard-feelings and internal strife. By this movement the Association will antagonize other journals, and the friends of such journals are discriminated against, and the entering wedge for disruption and total ruin of the Association, will be admitted. And if such continues to be the policy of any Association, the sooner it dies—the better both for the medical press and the profession.

### THE AMERICAN MEDICAL ASSOCIATION.

This national medical body held its annual meeting at New Orleans, commencing on 28th of May last. It was the 36th meeting of the Association, and was largely attended. The hall was admirably adapted for the Association, and the new arrangement for registration was a decided improvement, and avoided confusion and saved much time in organizing the body.

The Association met promptly at 11 o'clock, A. M., and was called to order by Dr. Samuel Logan, Chiarman of the Committee of Arrangements.

A very appropriate and impressive prayer was offered by the Rev B. M. Palmer, D.D., after which the address of welcome was made by Dr. Samue Logan. His remarks were exceedingly appropriate and eloquent, eminently characteristic in its warmth and sentiments of the hospitality of the great Southern city of New Orleans.

Next came the Annual Address by the President, our distinguished Georgian, H. F. Campbell, of Augusta. We have not space to copy any portion of this grand and noble address, which we listened to with profound interest and attention. It was not only instructive in the ability of its utterances, but touching to the heart in the beauty and pathos of its sentiments.

After the address the business of the Association was taken up and conducted with more than the usual harmony and satisfaction.

The local entertainments, both by the profession and citizens, were of a superior order, and just what was expected of the warm hearted and highly cultivated people of the crescent city. There was only one regret which we have to express, and that was our inability to meet with, and shake with cordial hand, the entire number of professional brethren there assembled. The time was too short, and the diversion made by the great Exposition was too strong, to allow us opportunity for this pleasure to the extent desired.

The business papers presented to the various sections of the body were fully up to the average standard in respect to interest and ability, and we propose, from time to time, to cull from them items of progress and practical interest for our readers.

Those desiring the papers and proceedings in detail, can procure them by ordering the Journal of the American Medical Association, published by the venerable editor of the Association, Prof. N. S. Davis, M.D., LL.D.

We learn that a meeting of the Association of the American Medical Editors took place as usual on the day preceding the opening of the American Medical Association, but we have not as yet seen a report of its action. It is unfortunate that this meeting of the editors is appointed so early, as few can be present to attend it. It were better that one evening be set apart for this purpose and the doors be opened to such members of the general association as desire to attend. If this plan were adopted it would impart renewed life and impetus to the Editors Association, which ought to be a great and useful body, but which under the present arrangement is attended with little interest and accomplishes little or no good. Under the plan proposed, the editors in council would hold a relation to the main body somewhat like to that of the Senate to the House of Representatives in the U. S. Congress, being in a position to overlook, revise and discuss through the medical press all that is done with an eye to the grand good of the profession throughout the Union. The true and faithful journalist should be an exponent of medical ethics and a defender against wrong in all controversies that arise, and the Association, as an organized brotherhood, should see to it that the right be vindicated and the wrong opposed in every section of our country. The ethics, in its essence, simply means the golden rule, "to do unto others as we would be done by," and is as binding on one part of the country as another, and each part is bound to protect the other parts if assailed or injured. This is not only ethics, but it is justice and patriotism, and is wholly incompatible with that selfishness and narrow minded conduct so often found in our medical societies, in the management of our colleges, and sometimes, we regret to say, with editors of medical journals.

All must admit that our remarks are true and in accordance with correct principle, and failing to appreciate this truth on the part of medical journalists, and to let truth and principle guide in every case, is the cause of so much wrong, and so much error in our ranks, and a great drawback to the dignity and influence of the profession and to the cause of medical progress in the world.

It in nowise conflicts with the views here expressed, that every Association, every journal, every college and every individual should, by fair, honorable and legitimate methods, work for and promote their several interests, but in any and every case where the principles of honor and justice are violated, the entire strength and influence of the ethical journals, and of the true men of the profession throughout the Union, should be brought to bear against the wrong and in the advocacy of the right.

In our last we published the names of the officers for the ensuing year. The President elect, Dr. Wm. Brodie, at the close of the session, was conducted to the stand by Drs. Toner and Richardson, and made a short but handsome address, which was highly appreciated by the members of the body. Dr. Brodie is one of the oldest members and most earnest workers of the Association. We doubt not he will dignify the position, and will be patriotic in sentiment and liberal in action to all sections of our common country.

Dr. Campbell, the retiring President, in warm and enthusiastic language, thanked and complimented the members upon their good order and courteous attention during the discussions, and adjourned the body to meet in St. Louis, Mo., on the first Tuesday in May, 1866. P.

NEW ADVERTISEMENTS.—We invite attention to the following new advertisements in this issue of our journal, to-wit: Louisiana Medical College, and Ridge's Food, by the Chatterton Publishing Co., and the new advertisement of Peacock Chemical Co; also see advertisemet of Bellevue Hospital Medical College.

IS IT SO ?—The editor of the Phys. Investigator says that he has glanced over his accounts with the medical profession and is almost inclined to think that they are the poorest payers, taking them all together, of any class of men. Please disabuse his mind and send on your money for past dues and for the present year. Turn over a new leaf and pay for your medical journals at once.

CLINICAL THERMOMETRE.—We have received one of those excellent Clinical Thermometres, styled, *Reliable Clinical Thermometre*, from the house of A. M. Leslie Surgical Instrument Co., 204 North Broad Street, St. Louis. We are very much pleased with this style of thermometre and recommend it as a splendid instrument, and sold at the moderate price of $2.00.

